# Efficacy and Safety of Novel Oral Anticoagulants in Atrial Fibrillation: A Systematic Review

**DOI:** 10.7759/cureus.46385

**Published:** 2023-10-02

**Authors:** Prithvi Basu Roy, Vitrag N Tejani, Sukhmeet S Dhillon, Nanush Damarlapally, Tanusha Winson, Nia Uswanti Binti Usman, Binay K Panjiyar

**Affiliations:** 1 Medicine, KPC (Kali Pradip Chaudhuri) Medical College and Hospital, Kolkata, West Bengal, India, Kolkata, IND; 2 Medicine, Parul Institute of Medical Sciences and Research, Vadodara, IND; 3 Internal Medicine, Baba Farid University of Health Sciences, Faridkot, IND; 4 Health Sciences, Houston Community College - Coleman College for Health Sciences, Houston, USA; 5 Internal Medicine, AIMST University, Bedong, MYS; 6 Internal Medicine, Universitas Brawijaya, Malang, MYS; 7 Global Clinical Scholars Research Training, Harvard Medical School, Boston, USA; 8 Internal Medicine, California Institute of Behavioral Neurosciences & Psychology, Fairfield, USA

**Keywords:** new oral anticoagulants (noacs), afib, direct oral anticoagulants (doac), atrial fibrillation (af), efficacy and safety

## Abstract

In recent times, novel oral anticoagulants (NOACs)/direct oral anticoagulants (DOACs) have emerged as an alternative to the traditionally used Vitamin K oral antagonists (VKA) like warfarin for the treatment of atrial fibrillation (AF). This systematic review and meta-analysis aims to evaluate the efficacy and safety of NOACs in patients with AF and, thus, the related thromboembolic risks and sequelae. Of the 131 published articles we examined, 11 were included in an in-depth systematic review. The articles we reviewed were from the past ten years, from 2013 onward. The analysis derived the efficacy and safety of NOACs in patients with AF and also included different patients' baseline characteristics and subgroups. This systematic review reiterates previous research findings of superior efficacy and safety of the use of NOACs in the AF population and also illuminates certain head-to-head comparisons of individual NOACs with warfarin. It digressed into subgroups of patients with different baseline characteristics to provide evidence and support the existing guidelines for the use of NOACs in the treatment of AF. Overall, there is marked efficacy and safety of NOACs in patients with AF, be they elderly or Asian, with decreased renal function, or with other comorbidities. Adherence to NOACs was also satisfactory. Despite such a review, there needs to be more research on vast subgroups and also on reversal antidotes like andexanet alfa and idarucizumab, as well as more head-to-head analysis between NOACs over a long duration of study, which would provide more answers and pinpoint reasons as to the differences that exist between demographics and subgroups in the usage of NOACs.

## Introduction and background

Atrial fibrillation (AF) is characterized by a quivering, fluttering, irregular heartbeat. It is a type of tachyarrhythmia. The complications associated with it are cerebrovascular accident (CVA), left atrial appendage thrombus, heart failure, thromboembolism, cognitive decline, etc. The occurrence and frequency of AF is on the rise worldwide. According to the Framingham Heart Study, the prevalence of AF has increased threefold in the last 50 years. As of 2016, the Global Burden of Disease project assessed a total prevalence of 46.3 million throughout the world globally. The likelihood of a Caucasian man or woman developing AF in their lifetime was 1:3 and 1:5, respectively, that of Black individuals in 2014 [1]. Formerly, Vitamin K antagonists like acenocoumarol, phenprocoumon, and warfarin were used to anticoagulate patients with AF [2]. The need for regular monitoring by the international normalized ratio (INR), dietary and drug Interactions, narrow therapeutic window, delayed onset and offset, bleeding risks including gastrointestinal (GI) and intracranial hemorrhage (ICH), and patient compliance and inconvenience were the reasons for the need to shift to novel oral anticoagulants (NOACs) in this patient group [3]. NOACs, or direct oral anticoagulants (DOACs), are a class of drugs that is the new alternative to vitamin K antagonists.

NOACs were developed to combat the drawbacks and limitations of older drugs [4]. These drugs are used to treat and prevent conditions like stroke, deep vein thrombosis (DVT), pulmonary embolism, etc. The main advantages of using this class of drugs are predictable dosing, a simplified regimen, fewer drug and food interactions, rapid onset and offset, and lower bleeding risk. However, these have potential drawbacks such as cost, unavailability of antidotes in case of bleeding, and specific dosing adjustments for patients with kidney dysfunction.

This systematic review aims to synthesize the existing body of literature, including randomized control studies (RCTs), and observational and other studies to evaluate the comparative effectiveness and safety of NOACs in patients with AF. This review seeks to inform clinical decision-making, guide treatment strategies, and identify potential gaps in evidence that require further investigation. Through a rigorous analysis of collected data, this review tries to shed light on certain difficult questions, including the relative efficacy and outcome of different classes of NOACs such as stroke and bleeding risks, and the influence of NOAC therapy on patient outcomes and quality of life. This review aims to contribute to the ongoing refinement of guidelines and recommendations for anticoagulation in AF patients.

## Review

Methods 

**Figure 1:**
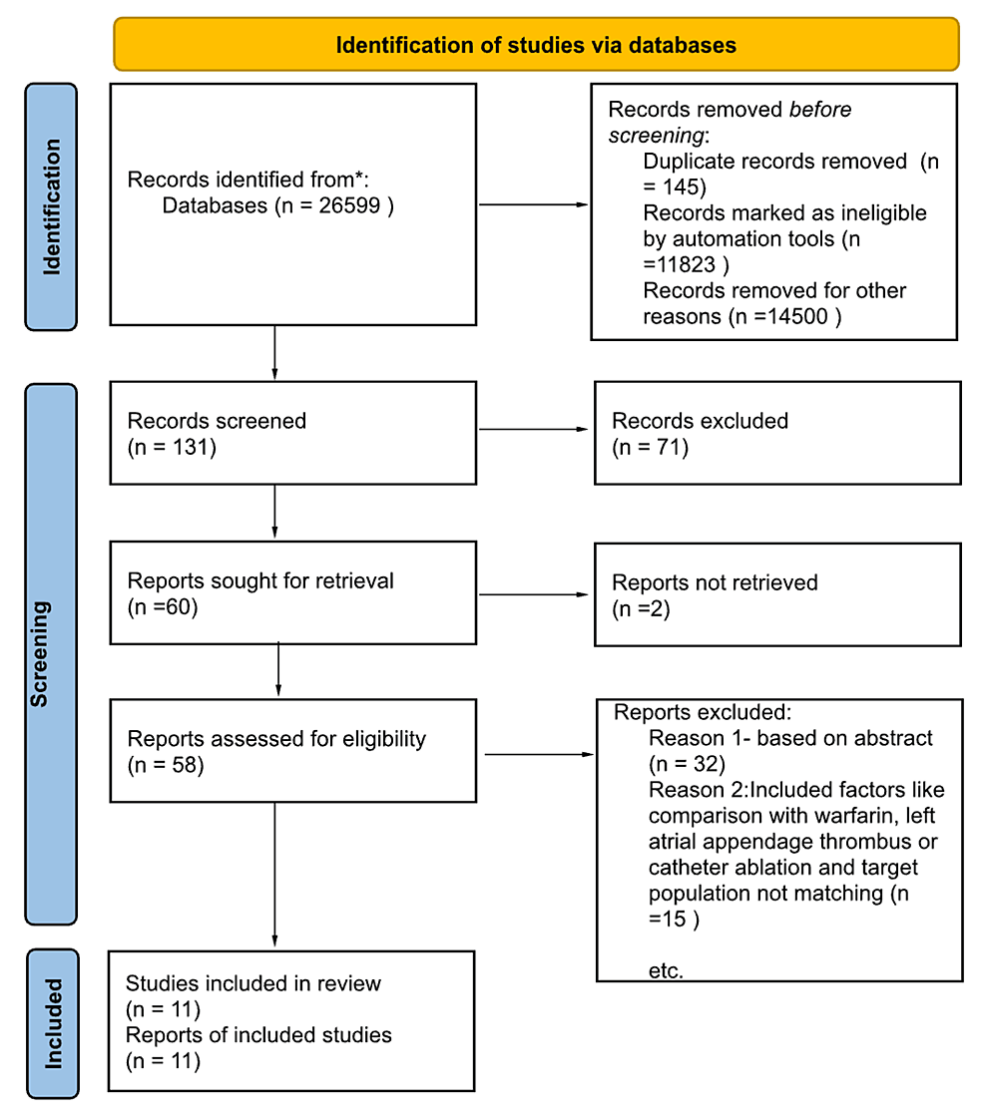
PRISMA flow diagram illustrating the search strategy and study selection process PRISMA: Preferred Reporting items for Systematic Reviews and Meta-Analyses

This review focused on clinical studies concerning the use of NOACs in patients with AF. The review followed the guidelines for Preferred Reporting Items for Systematic Reviews and Meta-Analyses (PRISMA) for 2020, as given in Figure 1, and only used data collected from published papers, eliminating the need for ethical approval. 

Systematic Literature Search and Study Selection

We conducted a thorough search for relevant publications in PubMed (including MEDLINE (Medical Literature Analysis and Retrieval System Online)) and Google Scholar databases. Apart from studies mentioned in review papers, editorials, and commentaries, we had a list of abstracts that were independently reviewed for inclusion using specific criteria.

Inclusion and exclusion criteria: We established specific criteria for including and excluding participants to achieve our study goals. Our criteria can be summarized in Table 1. The inclusion criteria were the use of NOACs and a clearly defined clinical cohort in the study, which is patients with AF. We excluded animal studies and publications that considered the efficacy and/or safety of NOACs in AF patients undergoing catheter ablation of the left atrial appendage, defibrillator implantation, post-cardiac transplant, chemotherapy, or having significant prominent comorbidities, which could restrict the clinical cohort of patients that we are wanting to study. Four reviewers conducted a dual review, and disagreements were resolved through discussion.

**Table 1:** Exclusion and inclusion criteria adopted during the literature search process ^*^The PICO criteria utilized were: (i) Population, human adults of age >= 19 years having atrial fibrillation; Intervention,  treating the  study population with NOACs; Comparison group, compared with placebo; Outcome, decreased risk of stroke/cerebral vascular accident/thromboembolic manifestations/myocardial infarction/less adverse effects like GI bleeding /toxicity PICO: Population, Intervention, Control, and Outcome; NOACs: novel oral anticoagulants

Inclusion criteria	Exclusion criteria
Studies done only on humans.	Studies done on animals.
The text of the study is in English.	Non-English text
Studies between the time span of 2013-2023	Any studies conducted before 2013
Age of population under study >=19 years (Adults)	Age of population <19 years
Gender: both male and female	Papers that needed to be purchased
Free full texts	
Studies filtered by PICO criteria *	

Search Strategy

The search was conducted on PubMed (including MEDLINE) and Google Scholar databases, using relevant keywords, such as Efficacy and Safety, AF, Afib, NOACs, and DOACs. The medical subject heading (MeSH) approach for PubMed (including MEDLINE) and Google Scholar, as detailed in Table 2, was employed to develop a comprehensive search strategy.

**Table 2:** Search strategy, search engines used, and the number of results displayed NOAC: novel oral anticoagulants; DOAC: direct oral anticoagulants; VKA: vitamin K antagonists; AF/A fib: atrial fibrillation

Search	Database	Results	Date
(("Treatment Outcome"[Mesh] OR Efficacy[tw] OR Efficacious[tw] OR Efficac*[tw] AND "Toxicity" [Subheading] AND "Factor Xa Inhibitors"[Mesh] ) OR (oral anticoagulants)) OR (stop* clot formation) AND "Atrial Fibrillation/drug therapy"[Mesh]	PubMed	5399	29/07/2023
(("Treatment Outcome"[Mesh] OR Efficacy[tw] OR Efficacious[tw] OR Efficac*[tw] AND "Toxicity" [Subheading] AND "Factor Xa Inhibitors"[Mesh] ) OR (oral anticoagulants)) OR (stop* clot formation) AND "Atrial Fibrillation/drug therapy"[Mesh] Filters: Free full text, Clinical Trial, Meta-Analysis, Randomized Controlled Trial, Review, Systematic Review, in the last 10 years, Humans, English, Female, Male, MEDLINE, Adult: 19+ years	PubMed	176	29/07/2023
((("Treatment Outcome"[MeSH Terms] OR "Efficacy"[Text Word] OR "Efficacious"[Text Word] OR "efficac*"[Text Word]) AND "Toxicity"[MeSH Subheading] AND "Factor Xa Inhibitors"[MeSH Terms]) OR (("mouth"[MeSH Terms] OR "mouth"[All Fields] OR "oral"[All Fields]) AND ("anticoagulants"[Pharmacological Action] OR "anticoagulants"[Supplementary Concept] OR "anticoagulants"[All Fields] OR "anticoagulant"[All Fields] OR "anticoagulants"[MeSH Terms] OR "anticoagulate"[All Fields] OR "anticoagulated"[All Fields] OR "anticoagulating"[All Fields] OR "anticoagulation"[All Fields] OR "anticoagulations"[All Fields] OR "anticoagulative"[All Fields])) OR ("stop*"[All Fields] AND "clot"[All Fields] AND ("formations"[All Fields] OR "metabolism"[MeSH Terms] OR "metabolism"[All Fields] OR "formation"[All Fields]))) AND "atrial fibrillation/drug therapy"[MeSH Terms] Filters: Free full text, Clinical Trial, Meta-Analysis, Randomized Controlled Trial, Review, Systematic Review, in the last 10 year	PubMed	552	31/07/2023
((("Treatment Outcome"[MeSH Terms] OR "Efficacy"[Text Word] OR "Efficacious"[Text Word] OR "efficac*"[Text Word]) AND "Toxicity"[MeSH Subheading] AND "Factor Xa Inhibitors"[MeSH Terms]) OR (("mouth"[MeSH Terms] OR "mouth"[All Fields] OR "oral"[All Fields]) AND ("anticoagulants"[Pharmacological Action] OR "anticoagulants"[Supplementary Concept] OR "anticoagulants"[All Fields] OR "anticoagulant"[All Fields] OR "anticoagulants"[MeSH Terms] OR "anticoagulate"[All Fields] OR "anticoagulated"[All Fields] OR "anticoagulating"[All Fields] OR "anticoagulation"[All Fields] OR "anticoagulations"[All Fields] OR "anticoagulative"[All Fields])) OR ("stop*"[All Fields] AND "clot"[All Fields] AND ("formations"[All Fields] OR "metabolism"[MeSH Terms] OR "metabolism"[All Fields] OR "formation"[All Fields]))) AND "atrial fibrillation/drug therapy"[MeSH Terms] Filters: Free full text, Clinical Trial, Meta-Analysis, Randomized Controlled Trial, Review, Systematic Review, in the last 10 years, Humans, English, Female, Male, Adult: 19+ years, MEDLINE	PubMed	176	02/08/2023
("Treatment Outcome"[Mesh] OR ("efficacy"[tiab] OR "effectiveness"[tiab])) AND ("Biomarkers, Pharmacological"[Mesh] OR "Toxicity"[MeSH Subheading] OR "Safety"[tiab]) OR ("Factor Xa Inhibitors"[Mesh] AND "Anticoagulants"[Mesh]) OR ("novel oral anticoagulants"[tw] OR "NOACs"[tw] OR "DOACs"[tw] OR "direct oral anticoagulants"[tw] OR "non-vitamin K oral anticoagulant"[tw]) AND ("Atrial Fibrillation"[Mesh] OR ("Atrial Fibrillation"[tw] OR "AF"[tw] OR "A-fib"[tw]))	PubMed	413	02/08/2023
(efficacy OR effectiveness) AND safety AND (Novel oral anticoagulants OR Direct oral anticoagulants OR DOACs OR NOACs OR non- vitamin k antagonist oral anticoagulants OR New oral anticoagulants) AND (atrial fibrillation OR AF OR A-fib)	Google Scholar	21200	03/08/2023
(efficacy OR effectiveness) AND safety AND (Novel oral anticoagulants OR Direct oral anticoagulants OR DOACs OR NOACs OR non- vitamin k antagonist oral anticoagulants OR New oral anticoagulants) AND (atrial fibrillation OR AF OR A-fib) filters - search date 2013-2023.	Google Scholar	14600	03/08/2023

Quality Appraisal

We used a variety of quality assessment tools to ensure the dependability of the papers we selected. For RCTs used in systematic reviews and meta-analyses, we used the PRISMA checklist and Cochrane bias tool assessment. Using the Newcastle-Ottawa Tool scale, non-RCTs were assessed. Using the Critical Appraisal Skills Programme (CASP) checklist, we evaluated the quality of qualitative studies in Table 3.

**Table 3:** Quality appraisal tools used PRISMA: Preferred reporting items for Systematic Reviews and Meta-Analyses

Quality Appraisal Tools used	Types of Studies
Cochrane bias Tool assessment	Randomized Control Trial (RCT)
Newcastle-Ottawa Tool	Non-RCT and Observational Studies
PRISMA Checklist	Systematic Reviews

Results

We extracted 26,599 articles from two targeted databases, PubMed and Google Scholar. Following a thorough evaluation of each manuscript using predetermined criteria and filters, 26,468 articles were excluded. We decided not to use 71 of the remaining 131 papers because they were duplicates or had inadequate titles and abstracts. The remaining 58 papers were carefully scrutinized, and we eliminated 47 more as their substance did not fulfill our inclusion requirements. The next step was a rigorous quality review of the 11 papers that remained, and all met our standards. Our last systematic review included these 11 papers. Table 4 gives a thorough explanation of each.

**Table 4:** Summary of the selected papers VTE: venous thromboembolism; ICH: intra cranial hemorrhage; SEE: systemic embolic event; VHD: valvular heart disease; GIB: gastrointestinal bleeding

Article Title and Journal	Country and Year	Author Names	Study Design	Conclusion	Database
1. Direct oral anticoagulants for stroke prevention in atrial fibrillation: treatment outcomes and dosing in special populations; Ther Adv Cardiovasc Dis	United States 2018	Stacy et al. [4]	Meta-analysis	Based on the outcomes of the major phase III randomized control studies, DOACs have been approved for the reduction of stroke and SEE risk in patients with NVAF. There are no therapeutic interactions for any of the DOACs with regard to stroke or SEE prevention in patient subgroups with elevated risk for stroke, according to the available secondary analyses. Overall, the findings of secondary analyses show that the DOACs' recommended dose regimens consistently inhibit clotting across a range of patient populations.	PubMed
2. Efficacy and safety of the novel oral anticoagulants in atrial fibrillation: a systematic review and meta-analysis of the literature; Circulation	Japan 2012	Dentali et al. [5]	systematic review and meta-analysis.	When compared to warfarin, NOACs reduced overall and cardiovascular mortality, stroke and SE, major bleeding and intracranial bleeding.	Google Scholar
3. Long-Term Treatment with Apixaban in Patients with Atrial Fibrillation: Outcomes during the Open-Label Extension following AVERROES; Thromb Haemost.	Canada 2021	Benz et al. [6]	Randomized control trial .	The annual incidence of hemorrhagic stroke, stroke with a systemic embolism, and severe bleeding remained at the same low levels as those seen during apixaban treatment in the first trial. The long-term efficacy and safety of apixaban in patients with atrial fibrillation are supported by these findings, which are based on a median follow-up period of three years.	PubMed
4. The daily practice of direct oral anticoagulant use in patients with atrial fibrillation; an observational cohort study; PLoS One	Netherlands 2019	Gulpen et al. [7]	Observational cohort study	The majority of patients in this observational trial maintained therapeutic on-therapy plasma levels under circumstances of generally strong adherence, experienced no thromboembolic events and only a small number of bleeding events, and remained within therapeutic plasma levels.	PubMed
5. Major bleeding with dabigatran and rivaroxaban in patients with atrial fibrillation: a real-world setting; Clin Appl Thromb Hemost.	United States 2014	Fontaine et al. [8]	retrospective electronic medical record (EMR) and chart review	In the study population, significant intracranial hemorrhage occurred at a decreased rate. Although there have been anecdotal cases of significant bleeding, the use of innovative oral anticoagulants in a clinical environment does not appear to increase the risk of major hemorrhage.	PubMed
6. Effectiveness and safety of non-vitamin K antagonist oral anticoagulants in Asian patients with atrial fibrillation; Stroke	Asia 2017	Cha et al. [9]	Systematic review and meta-analysis	In actual clinical settings, all three NOACs showed comparable risks of ischemic stroke and reduced risks of ICH when compared to warfarin in a high-risk Asian AF group. Only dabigatran and apixaban significantly reduced all-cause mortality.	Google Scholar
7. Effectiveness and safety of non-vitamin K antagonist oral anticoagulants in Asian patients with atrial fibrillation and valvular heart disease; Curr Med Res Opin	Taiwan 2021	Li et al. [10]	retrospective cohort study	Our research revealed that NOAC had a comparable risk of bleeding in people with AF and VHD and was just as effective as warfarin at avoiding ischemic stroke. Moreover, as compared to warfarin, NOAC decreased the risk of VTE, ICH, and death. Given the small number of patients in the current trial with severe VHD.	Google Scholar
8. The role of non-vitamin K antagonist oral anticoagulants in Asian patients with atrial fibrillation: A PRISMA-compliant article; Medicine (Baltimore)	Asia 2020	Liu et al. [11]	Systematic review and meta-analysis.	NOACs were at least as effective as warfarin, but more safe than warfarin in Asian patients with AF. Apixaban was superior to other NOACs for reducing SSE, while edoxaban showed a better safety profile than other NOACs	PubMed
9. Risk analysis of new oral anticoagulants for gastrointestinal bleeding and intracranial hemorrhage in atrial fibrillation patients: a systematic review and network meta-analysis; J Zhejiang Univ Sci B	China 2017	Xu et al. [12]	systematic review and network meta-analysis.	When compared to one another, the risks of GIB and ICH are unaffected by AF therapy with NOACs based on a sizable pooled patient population.	PubMed
10. Phase III studies on novel oral anticoagulants for stroke prevention in atrial fibrillation: a look beyond the excellent results; J Thromb Haemost	Milan 2012	Pengo et al. [13]	Review article	The significant Phase III clinical studies of NOACs (apixaban 5 mg bid and dabigatran 150 mg bid) for the prevention of stroke and peripheral embolism in patients with AF demonstrated that they are not inferior to warfarin and even superior.Apixaban ought to be the primary option for patients with dyspepsia or a history of gastrointestinal bleeding. Apixaban or rivaroxaban may be preferable in extremely old patients with deteriorating renal function, or warfarin in cases of severe renal insufficiency.	Google Scholar
11. Stroke prevention in atrial fibrillation: a clinical perspective on trials of the novel oral anticoagulants; Cardiovasc Drugs Ther	United States 2016	Morais et al. [14]	Review article	There is improved treatment persistence with NOACs compared to VKAs and most importantly, the effectiveness and safety of NOACs in real-world clinical practice are consistent with the results of phase III trials.	Google Scholar

Discussion

AF is the most common arrhythmia of clinical significance In adjusted models; it is associated with increased morbidity, especially stroke and heart failure, and increased mortality.

Previously, oral antiplatelets like aspirin and clopidogrel or vitamin K antagonists like warfarin or acenocoumarol, and phenprocoumon were used in the primary or secondary prevention of thromboembolic events [15]. Since the advent of NOACs in 2010, multiple landmark trials like AVERROES (Apixaban Versus Acetylsalicylic Acid to Prevent Stroke in AF Patients Who Have Failed or Are Unsuitable for Vitamin K Antagonist Treatment) study for apixaban [16], ROCKET AF (Rivaroxaban Once Daily Oral Direct Factor Xa Inhibition Compared with Vitamin K Antagonism for Prevention of Stroke and Embolism Trial in Atrial Fibrillation) trial for rivaroxaban [17], RE-LY (Randomized Evaluation of Long-term Anticoagulant Therapy) trial for dabigatran etexilate [18], and ENGAGE AF-TIMI 48 (Effective Anticoagulation with Factor Xa Next Generation in Atrial Fibrillation-Thrombolysis in Myocardial Infarction 48) trial for Edoxaban [19], American College of Cardiology, and European Society of Cardiology recommend the use of NOACs in the prophylaxis of thromboembolic events like stroke in our study population [20,21].

In this systematic review, we embark on a journey to evaluate the efficacy and safety of the NOACs in patients with different baseline parameters of patients with AF, be it valvular or non-valvular. We have digressed into different situations and demographics of the population with AF and tried to find the efficacy and safety profile and dosing of each individual NOAC and state its merits and demerits, which previous systematic reviews have either not compiled thoroughly or at that time head to head analysis and subgroup analysis were not available or studied upon.

One of the initial systematic reviews and meta-analyses on the same title as this study was conducted in 2012 by Dentali et al. [5]. The analysis, which included over 50,000 patients, showed that NOACs reduced total mortality, cardiovascular mortality, and the events of stroke or systemic embolism compared to vitamin K antagonists like warfarin. The study also found a significant reduction in ICH. Although there was no difference in the risk of myocardial infarction between NOACs and vitamin K antagonists.

The limitations of the study were that the definition of major or critical bleeding was heterogeneous between the studies considered and the baseline patient parameters and characteristics were different. This study at that time couldn't evaluate specific subgroups of patients or compare and contrast NOACs with each other or with warfarin-treated subgroups. The funnel plots also showed publication bias. To address the limitations of this study, we have tried to incorporate all the latest developments and findings in the literature and create an honest real-world perspective in Table 5.

**Table 5:** Table comparing some of the common parameters in the studies taken into the study. ^*^ Event rate of thromboembolic/hemorrhagic manifestations; ^n^ age of the population under study. HASBLED: hypertension, abnormal renal and liver function, stroke, labile INR, elderly > 65 years, drugs, and alcohol; CHA2DS2Vasc: congestive heart failure, hypertension, age > 75 years or older, diabetes, transient ischemic attack, previous stroke or thromboembolism, age 65-74 years, sex category (female)

Study	CHA2DS2Vasc Score	HASBLED	Event Rate *	Age** (in years)	Comments
Benz et al. [6]	3.4±1.5 s.d.	1.2±0.8 s.d.	1%( ischemic stroke) Hemorrhagic stroke – 0.3% Major bleeding -1.2 %	70	Open label extension study of apixaban
Gulpen et al. [7]	2.14	>=3	0.6% (major bleeding) 1.6 % (Minor bleeding)	75	Study measured plasma levels of NOACs over time.
Fontaine et al. [8]	3.46±1.3 (SD)	4.08±1.12 (SD)	0.5%	80.1	Major bleeding with Dabigatran and rivaroxaban .
Cha et al. [9]	3.51 (Rivaroxaban) and 3.6 (Dabigatran etexilate).	-	-	75	Asian population

AVERROES showed a clear benefit for apixaban over aspirin in patients with AF who were considered unsuitable for vitamin K antagonist therapy [22]. During the open-label extension of AVERROES conducted by Benz et al., apixaban was effective and had low rates of stroke or systemic embolism or hemorrhagic stroke (0.3% vs 1.2% in the double-blind study) and major bleeding during the follow-up period of three years [6]. The event rate of thromboembolic events was lower in the open-label study as compared to the double-blinded study, which is 1% vs. 1.6% per year. Analyses based on age, body weight, renal function, baseline CHA2DS2-Vasc (congestive heart failure, hypertension, age > 75 years or older, diabetes, transient ischemic attack, previous stroke or thromboembolism, age 65-74 years, sex category (female)) score, and a modified HASBLED (hypertension, abnormal renal and liver function, stroke, labile INR, elderly > 65 years, and drugs and alcohol) score showed efficacy of apixaban across these subgroups of patients with AF. Apixaban showed similar overall and cardiovascular mortality as compared to the original study indicating an older, high-risk patient population with many comorbidities. In the Aristotle trial, post-trial thromboembolic events rose due to a shift to warfarin [23].

To look at the efficacy of NOACs from a perspective of plasma concentration vs. effect and outcomes, Gulpen et al. did an observational cohort study on it [7]. In the elderly population with AF, due to comorbidities, multiple drug intake, and lack of adherence, conducting a study to evaluate the reduced and standard prescribing dose of NOACs and their plasma concentration over time was imperative. In 41% of patients using dabigatran and 14.4% of patients using rivaroxaban reduced treatment doses were prescribed. Both these drugs turned out to have similar plasma concentrations at standard and reduced doses, thereby possibly hinting at some of the recipients with the low dose having chronic kidney disease (CKD) or renal dysfunction hampering the excretion of the drug. It appeared that a significant proportion of patients started within the extreme values of upper and lower 20th percentiles and remained there throughout the one-year follow-up. In the study, there was no correlation found between higher values and negative outcomes; thereby, we can infer that one can safely switch between NOACs in the initial stage without adverse outcomes. Adherence to NOACs was moderate to high in the one-year follow-up with Morisky Medication Adherence Scale (MMAS), between 6-8.

The risk of major bleeding had to be seen in the real world as compared to RCTs on NOACs in patients with AF. Fontaine et al. conducted such a retrospective study where major bleeding was defined as bleeding into a critical organ or organ space, or other bleeding in the setting of transfusion of ≥2 units of packed red blood cells which differs slightly from the definition of critical bleeding used in major RCTs on NOACs [8]. Among patients who experienced a major bleed, the average age was 80.1 years (SD 7.62). The mean CHA2DS2-Vasc and HASBLED scores were 3.46 (SD 1.3) and 4.08 (SD 1.12), respectively. Nine patients (69.2%) were taking interfering drugs actively. In total, eight patients were concurrently receiving 81 mg/day of aspirin, and two patients were receiving long-term meloxicam therapy. One patient was on meloxicam as well as aspirin. This study showed a major bleeding rate of 0.5% (95%CI 0.23-0.77), which is quite similar to other prospective studies. Interacting medications like non-steroidal anti-inflammatory drugs (NSAIDs), antiplatelets, P glycoprotein inhibitors, old age and comorbidities, and high HASBLED and CHA2DS2-Vasc scores predispose these patients to these bleeding outcomes, which explains the anecdotal instances of these events as shown in this study.

Asian patients with AF are known to have different characteristics compared with non-Asian patients with AF [24]. Asian population is more susceptible to bleeding and has less likelihood of obtaining a therapeutic range INR with warfarin and Asians in general have a higher risk of having systemic embolism, CVA, and hemorrhagic stroke than non-Asians. In the study conducted by Cha et al. also, NOACs showed comparable effectiveness but better safety, mortality, and combined endpoints when compared to VKAs [9]. The NOACs were associated with no significant difference in risk of ischemic stroke but significantly lower risk of ICH when compared with warfarin. Rivaroxaban was not associated with lower mortality or lower risk for combined endpoints or lower risk of ICH than warfarin even in the elderly population > 75 years, the other two were. The prescribed dose of NOACs in the Asian population is less compared to non-Asians, partly owing to the lower BMI, high incidence of ICH in Asians, and these being prescribed to patients having comorbidities like hypertension, dyslipidemia, renal dysfunction, and those who are on multiple drug therapy.

NOACs used in AF patients in a setting of valvular heart disease (VHD), that is valvular AF, in an Asian population, were studied by Li et al. [25]. The risk of major bleeding increased in patients with VHD taking rivaroxaban but the risk of ICH was comparable to that of warfarin. In the meta-analysis, a reduction in the risk of developing ICH and major bleeding was demonstrated by all NOACs except rivaroxaban. The efficacy and safety of dabigatran have been proven to be inferior to warfarin in patients with mechanical heart valves in the report of the RE-ALIGN (Randomized Phase II Study to Evaluate the Safety and Pharmacokinetics of Oral Dabigatran Etexilate in Patients after Heart Valve Replacement) trial [26]. This study showed data that revealed differences in the effectiveness of different NOACs. the VTE risk was mitigated by dabigatran but the overall mortality and ICH risk were reduced by both dabigatran and rivaroxaban. NOACs showed comparable risk of ischemic stroke, transient ischemic attack (TIA), and GI bleeding to warfarin but reduced risk of VTE/ICH and overall mortality when compared to warfarin [10].

Further studies on Asian populations were conducted by Liu et al. based on data from either RCTs or real-world studies, for the prevention of systemic embolic events (SEE). The preferred sequence is apixaban followed by rivaroxaban then dabigatran then edoxaban, and finally warfarin, but for major bleeding and ICH preferred sequence is edoxaban followed by apixaban then dabigatran then rivaroxaban, and finally warfarin [11]. This study showed that edoxaban showed better safety profiles in major bleeding and ICH and a lower risk of ischemic stroke when compared to other NOACs. Apixaban had a lower risk of SEE compared with other NOACs.

Next is a study conducted by Stacy et al., which brings out results of NOACs used in AF patients under different subgroups and populations with risk factors for increased adverse outcomes [4]. Concomitant use of amiodarone or antiplatelet medication had no effect on the relative advantages of DOACs. There was no interaction between renal function and the efficacy and safety outcomes of DOACs. There was no interaction between the efficacy and safety results of DOACs and diabetes or hypertension, and elderly patients benefited from DOACs in a manner similar to that experienced by younger patients. Patients with a history of heart failure, stroke, or vascular disease were similarly treated with DOACs in terms of effectiveness and safety. Women with AF responded well and safely to DOACs. The study also recommended a dosing strategy in the patient subgroups [27-30].

Xu et al. took data from 20 randomized controlled trials with a total of 91,671 AF patients receiving anticoagulants, antiplatelet drugs, or placebo [12]. The review's findings demonstrated that, as compared to a placebo, aspirin+clopidogrel considerably increased the incidence of GI hemorrhage. There were no appreciable variations in GI bleeding risk among AF patients on aspirin, warfarin, or NOACs. Warfarin was discovered to significantly raise the risk of ICH in comparison to edoxaban 30 mg and dabigatran 110 mg. When compared to normal care or a placebo, NOACs did not raise the risk of ICH. NOACs were ranked as having the lowest risk of GI bleeding and ICH (apixaban 5 mg, dabigatran 110 mg, and edoxaban 30 mg) in the study. The authors concluded that when NOACs are used to treat AF patients with anticoagulants, the risks of GI bleeding/ICH are not higher than they are with other anticoagulants.

In the review article on phase III studies on NOACs for stroke prevention in AF authored by Pengo et al., it was seen that NOACs were not inferior to warfarin in the prevention of stroke and peripheral embolism in the study population and might even be superior, although the choice and dose of such NOACs depends on certain factors like age, comorbidities, drug interaction, and renal profile [13]. Dabigatran 150 mg bid or apixaban 5 mg twice daily may be considered in an elderly patient older than 65 years old with minimal bleeding risk and good renal function. In a similar case, dabigatran 110 mg or apixaban 5 mg twice daily may be the best option if the patient has moderate renal insufficiency (creatinine clearance 30-50 ml/minute) or a higher risk of bleeding. Rivaroxaban may be favored in high-risk elderly individuals with recent acute coronary syndrome, congestive heart failure, or a history of stroke. The above study also found that rivaroxaban is given only once a day, and may be explored in patients with anticipated low compliance. Dabigatran, on the other hand, might be taken into consideration in individuals taking several drugs due to its lower risk of drug interactions (no cytochrome metabolism). Apixaban ought to be the first option for people who have experienced past GI bleeding or dyspepsia. Warfarin may be used in cases of severe renal insufficiency or either apixaban or rivaroxaban may be recommended in extremely old patients with deteriorating renal function. 

The fact that NOACs have demonstrated enhanced efficacy and safety compared to warfarin in the prevention of stroke for patients with AF was reiterated in a review on stroke prevention in patients with AF on NOACs, by Morais et al. [14]. Dabigatran, rivaroxaban, apixaban, and edoxaban are examples of NOACs that have been shown to have a lower risk of bruising and intracranial bleeding. Individualized care is required, taking into account elements including age, renal impairment, and concurrent antiplatelet medication use. When prescribing NOACs, potential interactions with rhythm-controlling medications must be taken into account. A new field of study is focused on creating particular reversal medications for NOACs.

Future questions for researchers and gaps in research 

The generalizability of results may be impacted by the heterogeneity of study designs, which can introduce variations in techniques, patient demographics, and outcomes. Although some studies include follow-up periods of up to three years, the long-term effects of NOACs are not completely investigated beyond this time span. Results may be affected by drug interactions, comorbidities, and concurrent medication use. However, not all potential confounding factors can be taken into account, which may affect how NOACs are observed to work. Major bleeding and other adverse events may be defined differently in different studies, which might result in inconsistent reporting. Direct head-to-head comparison tests between NOACs are scarce. The need for reversal agents for NOACs is discussed; however, the accessibility and potency of these agents are not thoroughly examined. Longer follow-up times, uniform definitions, well-designed trials with diverse populations, and awareness of potential biases should all be used in future research to address these shortcomings.

Limitations 

We have limitations in our literature review. We focused on publications published over the last 10 years and restricted our analysis to a patient population that was at least 19 years old. Additionally, we only used free articles, and we only looked at English-language papers. More studies are required for definitive conclusions.

## Conclusions

Apixaban showed efficacy, low rates of side effects, and compatibility across several patient groupings, and revealed a clear advantage over aspirin in individuals who were not candidates for vitamin K antagonist therapy. Observational data demonstrated that decreased doses of NOACs were helpful in some patients, and plasma concentrations were consistent between standard and reduced doses, particularly in elderly AF patients. The study underscored the importance of personalized patient assessments when prescribing NOACs in AF because certain patients may have comorbidities, renal function, or use of counteracting medications. The study showed Asians' vulnerability to bleeding and revealed that NOACs could be as effective as warfarin while having better safety profiles. Although rivaroxaban exhibited inconsistent results in terms of bleeding risk, NOACs were typically efficacious and safe for individuals with valvular AF.

This review focused on selecting NOACs for stroke prevention, highlighting that NOAC selection and dose should take into account patient considerations such as age, comorbidities, and drug interactions. The study also shows that NOACs are more effective and safer than warfarin in preventing stroke in AF patients, indicating that, as compared to other therapies, NOACs did not raise the risk of GI bleeding or ICH. It also emphasized the various dangers associated with various anticoagulants. These discussions highlight the expanding body of research that supports the use of NOACs for AF treatment. They emphasize the necessity of tailored treatment approaches based on patient characteristics, as well as the need for continued research to improve dosing strategies, compare NOACs, and improve patient outcomes. 
